# A Homozygous Mutation in the *TUB* Gene Associated with Retinal Dystrophy and Obesity

**DOI:** 10.1002/humu.22482

**Published:** 2013-12-20

**Authors:** Arundhati Dev Borman, Laura R Pearce, Donna S Mackay, Kerstin Nagel-Wolfrum, Alice E Davidson, Robert Henderson, Sumedha Garg, Naushin H Waseem, Andrew R Webster, Vincent Plagnol, Uwe Wolfrum, I Sadaf Farooqi, Anthony T Moore

**Affiliations:** 1 Moorfield's Eye HospitalLondon, EC1C 2PD, UK; 2 Institute of OphthalmologyLondon, EC1V 9EL, UK; 3 University of Cambridge Metabolic Research Laboratories, Wellcome Trust-MRC Institute of Metabolic Science, Addenbrooke's HospitalCambridge, CB2 0QQ, UK; 4 Department of Cell and Matrix Biology, Institute of Zoology, Johannes Gutenberg, University of MainzMainz, Germany; 5 University College London Genetics InstituteLondon, WC1E 6BT, UK

**Keywords:** *TUB*, tubby, retinal dystrophy, obesity, cilia

## Abstract

Inherited retinal dystrophies are a major cause of childhood blindness. Here, we describe the identification of a homozygous frameshift mutation (c.1194_1195delAG, p.Arg398Serfs*9) in *TUB* in a child from a consanguineous UK Caucasian family investigated using autozygosity mapping and whole-exome sequencing. The proband presented with obesity, night blindness, decreased visual acuity, and electrophysiological features of a rod cone dystrophy. The mutation was also found in two of the proband's siblings with retinal dystrophy and resulted in mislocalization of the truncated protein. In contrast to known forms of retinal dystrophy, including those caused by mutations in the tubby-like protein TULP-1, loss of function of TUB in the proband and two affected family members was associated with early-onset obesity, consistent with an additional role for TUB in energy homeostasis.

Retinitis pigmentosa (RP) describes a genetically heterogeneous group of disorders characterized by night blindness, early peripheral visual field loss, and subsequent loss of central vision, leading to severe visual impairment. RP can be inherited in an autosomal-dominant, autosomal-recessive, or X-linked manner and mutations in over 60 different genes have been identified to date [den Hollander et al., 2010]. However, a significant proportion of RP remains genetically unexplained. RP may be seen in combination with obesity in Bardet–Biedl syndrome and Alstrom syndrome. These disorders, and other forms of RP, are referred to as “ciliopathies” as they are caused by mutations in genes important for the generation and maintenance of cilia [Waters and Beales, 2011].

The Tubby-like proteins (TUB, TULP1, TULP2, and TULP3) are a unique family of proteins that share a highly conserved C-terminal domain [Carroll et al., 2004]. They take their name from the tubby strain of obese mice in which a recessive, loss-of-function mutation in *Tub* causes retinal and cochlear degeneration, obesity, and insulin resistance [Coleman and Eicher, 1990; Kleyn et al., 1996; Noben-Trauth et al., 1996]. Recessively inherited mutations in *TULP1*, which is highly expressed in the retina and implicated in rhodopsin transport, are found in approximately 1% of patients with RP [Hagstrom et al., 1998; den Hollander et al., 2007]. However, disease-associated mutations involving other TUB family members have not been identified to date in humans.

An 11-year-old male from a consanguineous UK Caucasian family presented with deteriorating vision for 2 years. Visual acuity (VA) was 6/12 in the right eye and no perception of light (NPL) in the left eye. He had a bilateral myopic and astigmatic refractive error and retinal examination demonstrated a “blonde” fundus in the right eye and total retinal detachment with vitreous hemorrhage in the left eye. When reviewed at age 18 years, his best corrected VA was 6/9 in the right eye and NPL in the left eye. He had a bilateral myopic and astigmatic refractive error (right eye −1.25 dioptre sphere with −4.25 dioptre cylinder at 16°, and left eye −1.00 dioptre sphere with −4.00 dioptre cylinder at 170°). Hardy Rand and Ritler color vision testing of the right eye revealed a general disturbance of color vision affecting protan, deutan, and tritan axes. Funduscopy of the right eye demonstrated widespread retinal pigment epithelial atrophy, generalized retinal pallor, arteriolar attenuation, fine peripheral pigmentary mottling and white dots throughout the retina, with sparing of the macula (Fig.1A). There was no intraretinal pigment migration and no vitreoretinal interface abnormalities were identified on clinical examination or on optical coherence tomography (OCT) imaging in this eye. Funduscopy of the left eye showed a total retinal detachment. The visual field in the right eye, tested with Goldmann perimetry, was reduced to the central 15°.

**Figure 1:**
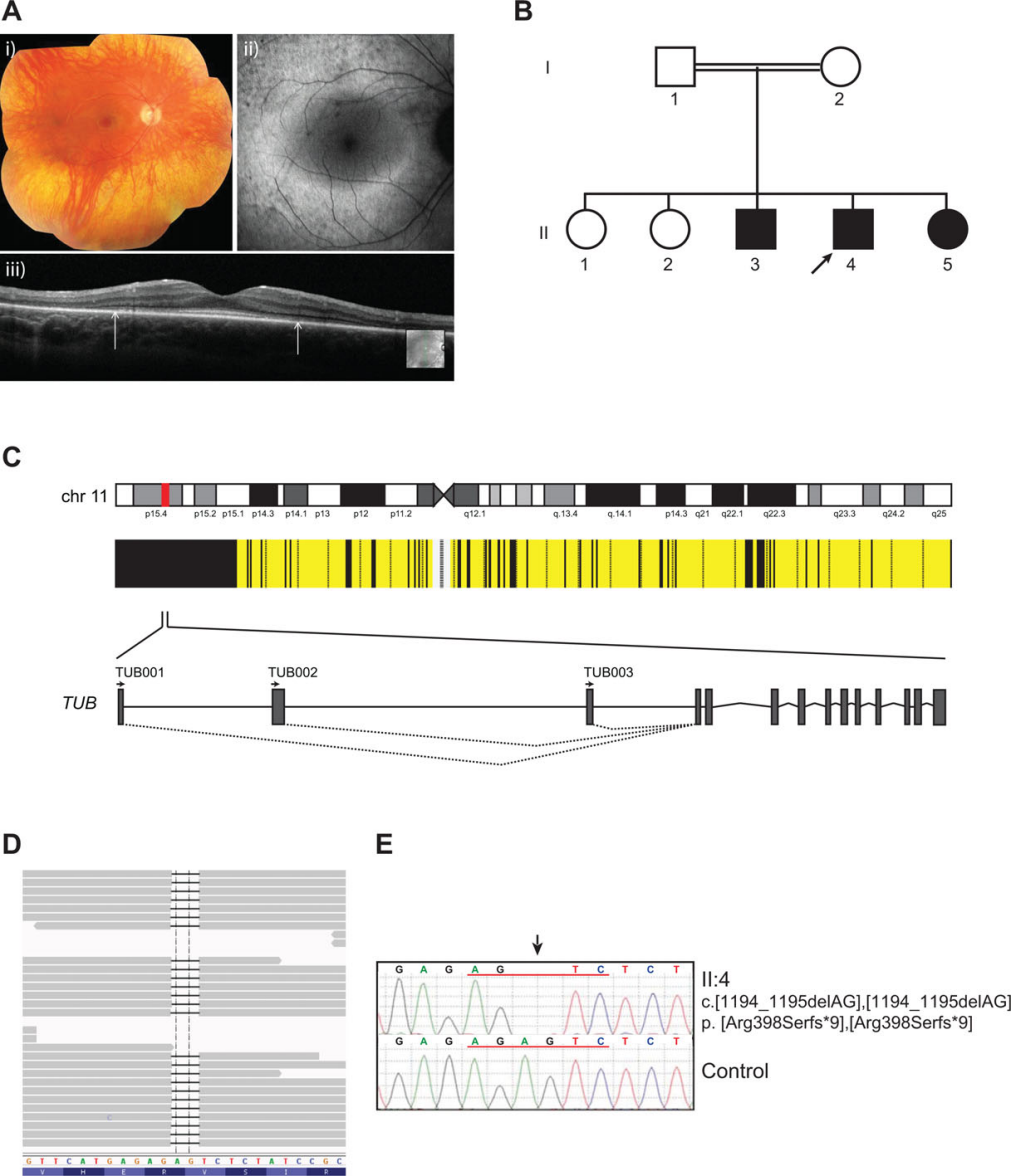
Identification of a homozygous frameshift mutation in TUB and clinical phenotype. A: Ocular images of proband. (i) Fundus photograph of right eye. (ii) Fundus autofluorescence of right eye. (iii) Spectral domain OCT image of right eye. Arrows mark the junction of preserved and nonpreserved IS/OS junctions. B: Pedigree of affected family. The proband (II.4) is indicated with an arrow. Solid symbols represent family members with retinal dystrophy (RD), open symbols unaffected family members. Circles represent females, and squares represent males. C: Schematic showing chromosome 11, blocks of homozygosity identified in the proband's DNA (generated using AutoSNPa), and the location and gene structure of *TUB*. Black and yellow bars indicate homozygous and heterozygous single-nucleotide polymorphism calls, respectively. The three splice variants that arise from the *TUB* gene are indicated (TUB001 (Ensembl ENST00000534099), TUB002 (Ensembl ENST00000305253 and TUB003 [Ensembl ENST00000299506]). D: Sequencing reads showing the homozygous mutation in *TUB* that was identified by exome sequencing (Integrative Genomics Viewer). E: Sequence chromatogram for the proband.

Full-field electroretinography (ERG) in the proband demonstrated a nonrecordable ERG in the left eye and severe loss of function in the right eye with absent rod responses and a small residual cone response, in keeping with a severe generalized rod-cone dystrophy. Retinal OCT imaging demonstrated preservation of the photoreceptor inner segment/outer segment (IS/OS) junction at the fovea, with loss of this layer in the parafoveal region (Fig.1A). This corresponded with the fundus autofluorescence (FAF) image, which demonstrated an annulus of hyperautofluorescence, commonly seen in patients with RP (Fig.1A). At the age of 18 years, the patient was obese with a body mass index (BMI) of 30 kg/m^2^, normal random glucose (5.0 mmol/L), HbA1c (39 mmol/mol), triglycerides (1.6 mmol/L), total cholesterol (4.3 mmol/L), and high density lipoprotein (HDL) cholesterol (1.1 mmol/L). There were no additional clinical features suggestive of Bardet–Biedl syndrome or Alstrom syndrome. Of note, no hearing problems were reported, although the patient had mild learning difficulties.

On the basis of the proband's consanguineous ancestry, his DNA was analyzed using homozygosity mapping [Lander and Botstein, 1987] (for details of experimental procedures, see Supp. Methods). Five chromosomal segments over 5 Mb were identified but none of these regions included known RP-associated genes (Supp. Table S1). Although four retinal disease-associated genes were found to be present in the 20.4-Mb region (*CTSD, TPP1, TEAD1*, and *USH1C)*, subsequent sequencing data demonstrated that the proband did not possess any disease-causing variants in these genes. Exon capture and high-throughput sequencing of the subject's DNA was then performed using solution-phase Agilent SureSelect 38-Mb exome capture. Average sequencing depth on target was 43 with 78.4% of the targeted region covered with a minimum read depth of 10. On the basis of the prior belief that RP-related mutations are rare; calls with minor allele frequencies greater than 0.5% in the 1000 genomes dataset were filtered. When prioritizing homozygous, presumed loss-of-function sequence alterations, we identified a homozygous frameshift variant in *TUB* (MIM #601197) c.1194_1195delAG, p.Arg398Serfs*9 (numbered according to Ensembl transcript ENST00000299506) (Supp. Table S2). This variant was located in the second largest region of homozygosity (Supp. Table S1) and was verified by Sanger sequencing (Fig.1C). The *TUB* variant identified in this study has been submitted to a *TUB*-specific database (www.lovd.nl/TUB). No potentially pathogenic variants were identified by exome sequencing in any genes currently known to be associated with RP or in any other retinal disease-associated genes (RetNet; http://www.sph.uth.tmc.edu/retnet).

The proband's older brother (II.3) and younger sister (II.5) were also found to harbor the *TUB* variant in the homozygous state (Supp. Fig. S1). They both had reduced vision (6/18 both eyes in the brother and 6/9 right, 6/12 left in the sister) and myopic astigmatic refractive errors (Supp. Table S3). The 21-year-old brother was not aware of any ocular problems but was found to have bilateral symmetrical widespread RPE atrophy, fine pigmentary mottling, and white dots throughout the retina (Supp. Fig. S1). There was hypofluorescent mottling along the vascular arcades but a normal foveal autofluorescence signal. The OCT demonstrated a preserved photoreceptor IS/OS layer at the fovea with outer retinal debris at the level of the RPE in the parafoveal region. The retina in the 9-year-old sister, who was asymptomatic, had bilateral widespread RPE atrophy but the pigmentary mottling was confined to the inferior retina. OCT and FAF imaging were normal. While the brother had a BMI in the normal range (23 kg/m^2^) at the age of 21 years, the sister was classified as obese at the age of 9 years, falling into the 98th centile for age and gender. It is not clear why the brother had a milder retinal phenotype and a normal BMI at the age of 21, although it is possible that environmental factors or other genetic modifiers may have influenced his phenotype. Unfortunately, data regarding his BMI in early childhood were not available and therefore it was not possible to ascertain whether obesity was a feature at a young age. Both the father (I.1), mother (I.2), and one unaffected sibling (II.2) were heterozygous for the variant and had BMIs of 30, 24, and 20 kg/m^2^, respectively (Supp. Table S3). The patient and family did not consent to further phenotypic studies such as smell acuity, auditory testing, and metabolic studies.

We screened *TUB* using Sanger sequencing in 96 additional probands with childhood-onset autosomal-recessive RP, where previous genetic investigations had not identified the causative gene, and in 55 patients with severe obesity and a variety of ocular phenotypes from the Genetics of Obesity Study (GOOS); no additional potentially pathogenic variants were found. In a previous study, sequencing of *TUB* in 294 subjects with recessive RP did not identify any causative mutations [Xi et al., 2006] and the *TUB* p.Arg398Serfs*9 variant was also not present in over 6,000 publicly available exomes (NHLBI exome variant server). In addition, only two frameshift variants, which are likely to result in complete loss of function of *TUB*, were found in 12,982 alleles (NHLBI exome variant server), indicating that homozygous loss of function of *TUB* is rare (estimated frequency <1/10^7^ individuals).

The *TUB* p.Arg398Serfs*9 variant falls within the highly conserved C-terminal tubby domain that is found in all TUB family members and results in a truncated form of TUB (Fig.1, Supp. Fig. S2). In fact, it would be predicted to lead to an even more severe truncation than that caused by the mutation originally identified in the tubby mouse. We found that when transfected into HEK293 cells, the p.Arg398Serfs*9 variant was expressed but at a reduced level compared with wild-type (WT) TUB (Fig.2B). While GFP–TUB WT was detected within the cytoplasm and at the plasma membrane, GFP–TUB p.Arg398Serfs*9 localized predominantly to the nucleus (Fig.2C). Subcellular fractionation also confirmed this mislocalization; the level of GFP–TUB p.Arg398Serfs*9 was reduced in the plasma membrane fraction but increased in the chromatin-bound fraction (Fig.2D). GFP–TUB p.Arg398Serfs*9, but not WT, was also found in the insoluble fraction of the pellet, potentially indicating that it may form aggregates within cells.

**Figure 2:**
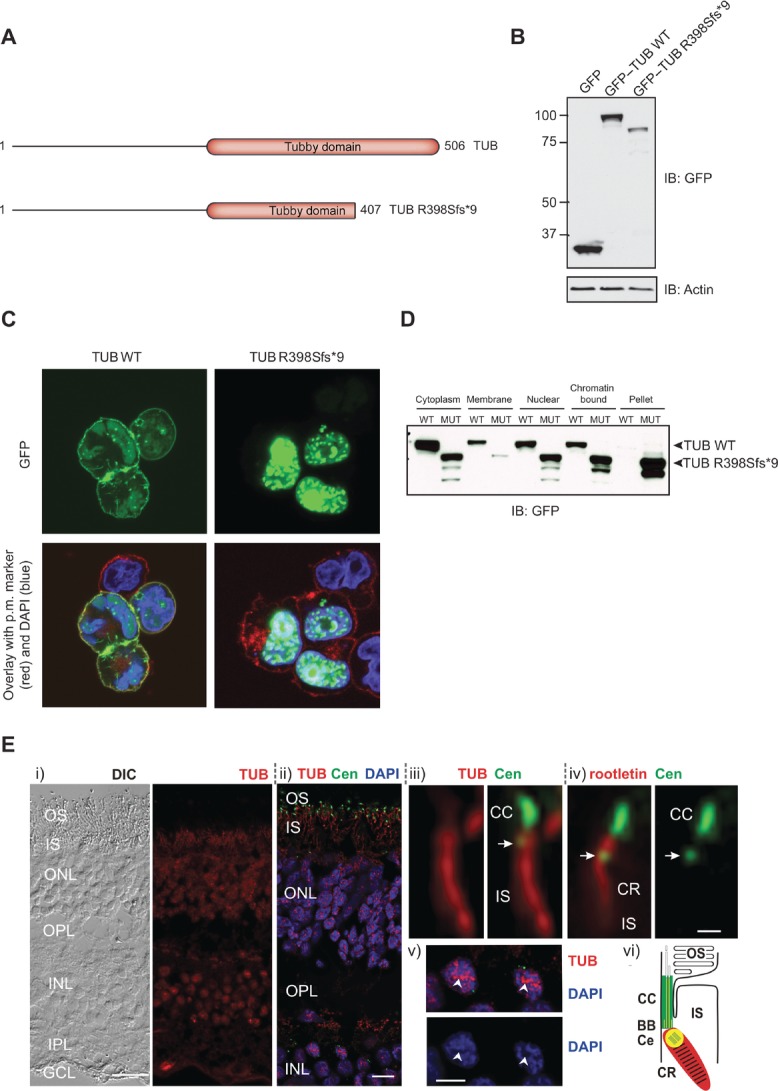
Functional characterization of TUB mutation. A: Schematic representation of the TUB WT and frameshift mutant proteins. B: HEK293 cells were transfected with the indicated constructs and resulting lysates subjected to immunoblotting with the indicated antibodies. C: Transfected HEK293 cells were fixed and localization of TUB proteins was determined by immunofluorescent staining. Green, GFP—Tub; red, plasma membrane marker (wheatgerm agglutinin alexa fluor 594); and blue, DAPI staining of nuclear DNA. D: Subcellular fractionation of HEK293 cells transfected with WT or mutant (MUT) GFP–TUB. E: Localization of TUB in human retinal cryosections. (i) Differential interference contrast (DIC) image and indirect immunofluorescence of anti-TUB (red). (ii) Merged images of double immunofluorescence of TUB (red), Centrin3 (Cen, green), and DAPI (blue). (iii and iv) High-magnification images of a single photoreceptor ciliary apparatus. Double labeling of (iii) TUB (red) and Centrin3 (green) and (iv) rootletin (red) and Centrin (green). Arrow indicates the centriole, CR, ciliary rootlet. (v) Labeling of TUB (red) and DAPI (blue) showing TUB localization in the euchromatin (arrowhead) of nuclei. (vi) Schematic cartoon of TUB (red) and Cen (green) localization in the photoreceptor ciliary region. TUB and Cen colocalize at the centriole (yellow). TUB and rootletin distribution is identical. BB, basal body; CC, connecting cilium; CE, centriole; IPL, inner plexiform layer; OS, outer segment; OPL, outer plexiform layer. Scale bars: (i)–(ii) 25 μm; (iv) 0.5 μm; and (v) 5 μm.

We also analyzed the distribution of TUB in the human retina by immunohistochemistry on cryosections using an antibody raised against the N-terminus of TUB. We found strong TUB expression in the nuclei of the ganglion–cell layer as well as the inner and the outer nuclear layer, and moderate staining in the inner segment of photoreceptor cells (Fig.2E). This contrasts with expression of the related Tub family member, TULP1, which is reported to be limited to photoreceptor cells only [Hagstrom et al., 1999, 2001; Ikeda et al., 1999]. Double immunofluorescence labeling of TUB and molecular markers of ciliary compartments, namely anti-centrin3 (connecting cilium, basal body and adjacent centriole marker [Trojan et al., 2008]) and anti-rootletin (photoreceptor ciliary rootlet marker [Yang et al., 2002]), revealed localization of TUB at the base of the photoreceptor cilium and the ciliary rootlet, projecting through the inner segment of the photoreceptor cell (Fig.2E).

The presence of TUB at the ciliary base and ciliary rootlet of photoreceptor cells is similar to that described in previous studies on TULP1 [Hagstrom et al., 1999, 2001; Ikeda et al., 1999]. Both compartments are thought to participate in the transport of ciliary cargo [Nachury et al., 2010; Gilliam et al., 2012] and there is growing evidence that TUB and other TUB family members play a role in the delivery of G protein-coupled receptors (GPCRs) to cilia [Mukhopadhyay and Jackson, 2011; Sun et al., 2012]. Defective rhodopsin transport is the likely mechanism by which photoreceptor degeneration occurs [Sun et al., 2012]. TULP-3 regulates GPCR trafficking to primary cilia through its interaction with components of the IFT-A complex, and mutation of the C-terminal domain in TULP-3 in mice results in reduced ciliary localization of the GPCR, MCHR1 [Mukhopadhyay et al., 2010]. While comparable studies are not possible in humans, it is plausible that the p.Arg398Serfs*9 variant, which disrupts the C-terminal domain, affects GPCR trafficking.

The presence of early-onset obesity in the proband and affected family members, in addition to RP, is consistent with the phenotype seen in *Tub* mutant mice, although this was not as severe as in other genetic forms of obesity. It has recently been shown that the targeting of two neuronal GPCRs, SSTR3, and MCHR1 to neuronal cilia is defective in *Tub* mutant mice [Sun et al., 2012]. The observation that MCHR1 localization is disrupted is of particular interest as it is involved in the regulation of energy intake and expenditure. In addition, TUB appears to be involved in mediating the effects of insulin and leptin within the hypothalamus [Prada et al., 2013]. Previous studies in mice have demonstrated that Tub is expressed in regions of the hypothalamus involved in feeding behavior and energy homeostasis [Sahly et al., 1998; Ikeda et al., 2000], and we also observed widespread TUB expression in human brain regions including the hypothalamus, as well as the retina (data not shown). Although TULP1 is also expressed in the same hypothalamic regions as TUB, obesity has not been reported in other families with *TULP1*-associated RP nor in *TULP1*^−/−^ mice [Lewis et al., 1999; Ikeda et al., 2000]. It is possible that this is due to discrete differences in their expression; previous reports have shown that in hypothalamic neurons TUB immunoreactivity is detected in the nucleolus of the nucleus, whereas TULP1 is only observed in distinct structures within the nucleus. However, further investigation will be necessary to fully understand the mechanisms underlying the shared and disparate phenotypes caused by mutations in *TUB* family members.

In summary, we describe a new ciliopathy characterized by retinal dystrophy and early-onset obesity due to a homozygous variant in *TUB*. Although further studies will be needed to fully elucidate the molecular functions of TUB, it is striking that the *TUB* variant reported here and all *TULP1* mutations described to date, disrupt the C-terminal domain found in all TUB family members. Further studies will be needed to test whether, as in mice, human TUB plays a role in the trafficking of GPCRs to cilia and in the modulation of signaling by hypothalamic GPCRs involved in energy homeostasis.

## Supporting Information

Disclaimer: Supplementary materials have been peer-reviewed but not copyedited.
