# Supplementary material for: A Homozygous Mutation in the TUB Gene Associated with Retinal Dystrophy and Obesity
Source: Hum Mutat. 2013 Dec 20;35(3):289–93. doi: 10.1002/humu.22482 (PMC4284018; doi:10.1002/humu.22482)
Supplement: Supplementary file 1 — Supplementary information [file humu0035-0289-sd1.pdf]

# **Supp. Methods**

## Clinical studies

Clinical examination included measurement of the best-corrected monocular visual acuity (VA) refractive status, slit-lamp biomicroscopy and funduscopy. Colour fundus photography using a Topcon TRC 501A retinal camera (Topcon Corporation, Tokyo, Japan), optical coherence tomography (OCT) using a Heidelberg SPECTRALIS® Spectral domain OCT scanner (Heidelberg Engineering, Dossenheim, Germany) or STRATUSOCT model 3000 scanner (Zeiss Humphrey Instruments, Dublin, CA, USA), and retinal autofluorescence (AF) imaging using a confocal scanning laser ophthalmoscope (Zeiss Prototype; Carl Zeiss Inc., Oberkochen, Germany) were performed in all patients. Colour vision assessments were performed using Ishihara pseudoisochromatic plates or the Hardy Rand and Rittler (HRR) pseudoisochromatic colour plates ($4^{th}$ Edition, Richmond Products Inc., USA). Goldman kinetic perimetry was performed where possible. Electrophysiological assessment including full-field ERG and pattern ERG (PERG) was performed incorporating the recommendations of the International Society for Clinical Electrophysiology of Vision (Holder, et al., 2007; Marmor, et al., 2009).

## Genetic Studies

The study followed the tenets of the Declaration of Helsinki and was approved by the Moorfields and Whittington Hospital local ethics committee. Patients and their parents for those under 16, provided written informed consent. Genomic DNA was extracted from peripheral blood lymphocytes using conventional methodologies and genome-wide single nucleotide polymorphism (SNP) microarray analysis was performed using the Affymetrix SNP Array 6.0. Homozygous regions were identified using AutoSNPa software (Carr, et al., 2006). Whole-exome sequencing was performed using the Agilent SureSelect38 Mb Human All Exon Kit and the HiSeq2000 sequencer (Illumina). Reads were aligned to the hg19 human reference sequence using Novoalign (Novocraft, www.novocraft.com) version 2.05. The ANNOVAR tool (OpenBioinformatics) was used to annotate SNPs and small insertions/deletions. ExomeDepth (Plagnol, et al., 2012) was used to call CNVs. The *TUB* coding regions were directly Sanger sequenced from PCR amplimers. *TUB* cDNA is numbered according to Ensembl transcript ENST00000299506. The *TUB* variant identified in this study has been submitted to the TUB-specific database (www.lovd.nl/TUB).

**Functional studies**

Full-length TUB (NM_177972.2) was PCR-amplified from a human brain cDNA library (Clontech), cloned into pEGFPC1 and mutant TUB (R398Sfs*9) prepared by site-directed mutagenesis. HEK293 cells were transiently transfected using the polyethylenimine method. 36hrs post-transfection, cells were stained with the plasma membrane marker wheat-germ agglutinin and fixed with 4% paraformaldehyde. Cells were permeabilised with 0.1% TBS-T, mounted on slides using Vectashield containing DAPI and visualized with a ZEISS LSM510 meta confocal microscope using Zen 2011 software. For subcellular fractionation, transfected cells were trypsinised, centrifuged at 500xg and fractionation performed using the ThermoScientific subcellular fractionation kit.

**Immunohistochemistry on human retinal cryosections**

Eyes from a human donor were obtained from the Department of Ophthalmology, University Medical School, Mainz, Germany. The guidelines to the declaration of Helsinki were followed. Human retinal cryosections were cryofixed in melting isopentane and cryosectioned as described elsewhere (Overlack, et al., 2011). Cryosections were incubated subsequently with 0.01% Tween 20 in PBS and after three PBS washes sections were incubated with blocking solution (0.5% cold-water fish gelatin plus 0.1% ovalbumin in PBS) followed by overnight incubation with primary antibodies (mouse anti-centrin3 (Trojan, et al., 2008), rabbit anti-tubby (Proteintech Group), rabbit anti-rootletin (Yang, et al., 2002)), diluted in blocking solution at 4°C. PBS washed cryosections were incubated with secondary antibodies conjugated to Alexa 488 and Alexa 555 (Molecular Probes) in blocking solution and 4',6-diamidino-2-phenylindole (DAPI) (Sigma-Aldrich) in PBS for nuclei staining. After PBS washes sections were mounted in Mowiol 4.88 (Carl Roth GmbH) and analysed on a Leica DM-6000B microscope (Leica Wetzlar, Germany). Images were obtained with a charge-coupled device camera (DFC 360FX, Leica,) and processed with Adobe Photoshop CS (Adobe Systems, San Jose, USA).

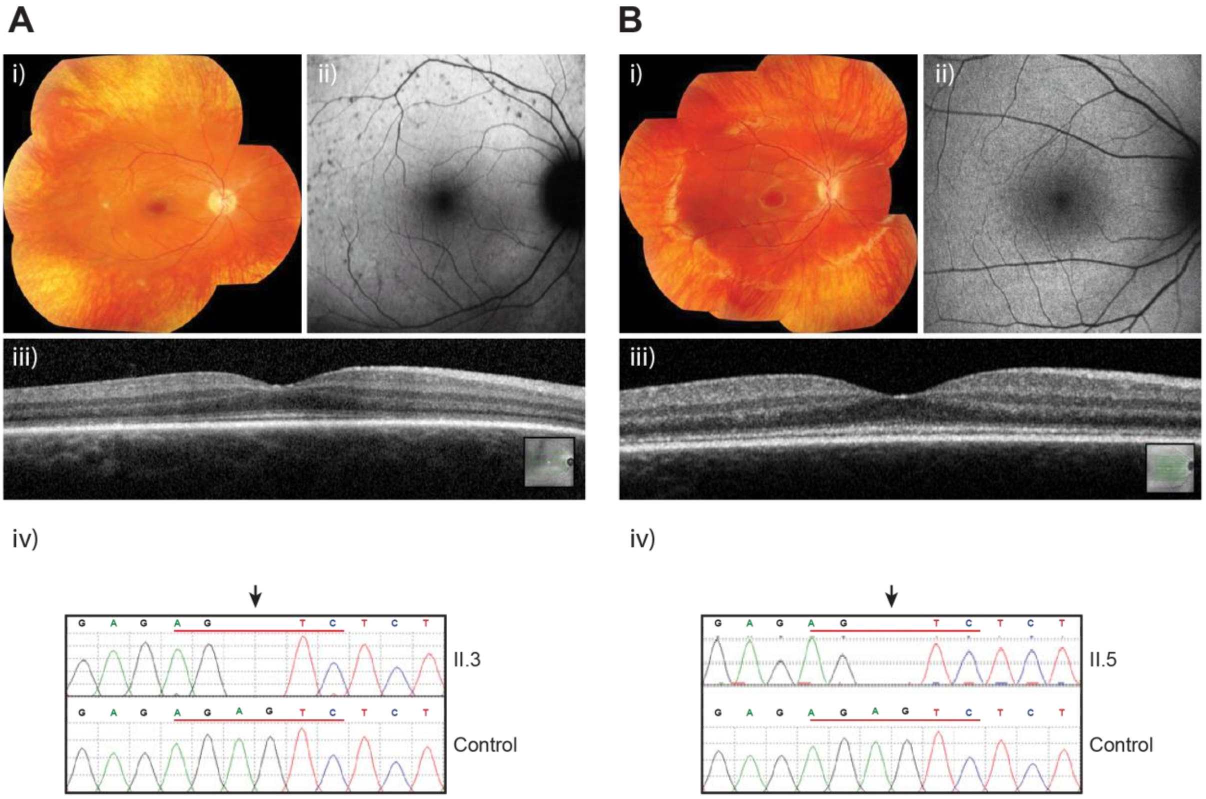


**Supp. Figure S1.** Clinical features of affected family members. **A)** Ocular images of older brother (II.3) and **B)** younger sister of proband (II.5). i) Fundus photograph of right eye, ii) Fundus autofluorescence of right eye, iii) Spectral domain optical coherence tomography (sd-OCT) image of right eye. iv) Sequence chromatogram showing the *TUB* c.1194-1195delAG, p.Arg398Serfs*9 variant.

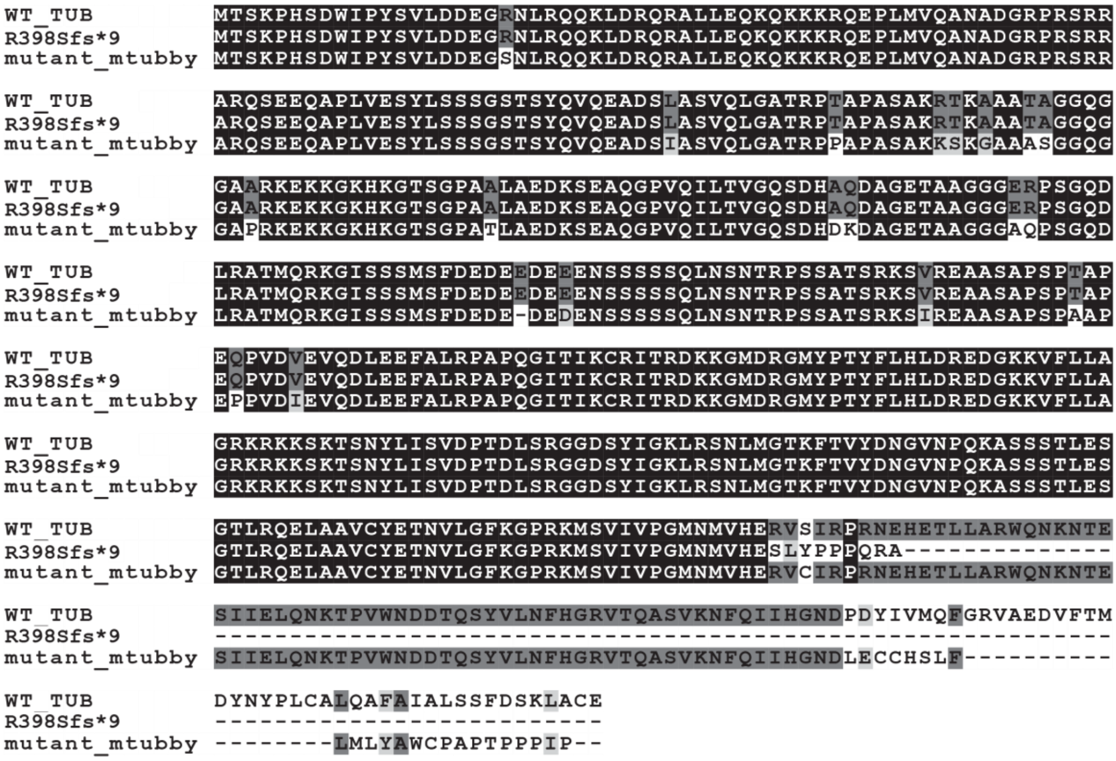


**Supp. Figure S2.** Alignment of wildtype and mutant human TUB with the mouse *tubby* mutant. The amino acid sequence of wild-type human TUB (Ensembl ENSP00000299506) is shown together with that of the truncated forms of TUB that result from the R398Sfs*9 variant and that originally identified in the *tubby* mouse (mutant-mtubby). Black shading indicates completely conserved residues, dark grey shows partially conserved residues, whilst light grey indicates similar residues.

**Supp. Table S1. Regions of homozygosity identified in *TUB* proband**

| Chromosome | From | To | Size (bp) | Retinal disease-associated genes |
|---|---|---|---|---|
| 5 | 53,911,084 | 76,517,333 | 22,606,249 | |
| 11 | 0 | 20,378,545 | 20,378,545 | *CTSD, TPP1,TEAD1, USH1C* |
| 14 | 31,647,241 | 40,881,971 | 9,234,730 | |
| 14 | 47,354,696 | 54,750,812 | 7,396,116 | |
| 15 | 93,245,168 | 99,611,746 | 6,366,578 | |

Genotype of proband was generated using the Affymetrix Genome-Wide Human SNP Array 6.0 (Affymetrix, Santa Clara, CA, USA). Regions of homozygosity were identified using AutoSNPa software (Carr, et al., 2006) and confirmed using an alternative method previously described (Sergouniotis, et al., 2011). Regions of homozygosity were considered significant if > 5 Mb. Regions of homozygosity were interrogated for the presence candidate retinal disease genes; 4 retinal-disease associated genes were found to be present within one of the homozygous regions. Subsequent exome sequencing data demonstrated that the proband did not possess any disease-associated variants in these given genes.

**Supp. Table S2. Prioritisation of variants identified by exome sequencing of DNA from *TUB* proband**

| | **Total** |
|---|---|
| All exonic variants | 16,990 |
| Total rare[a] nonsynonymous variants | 641 |
| Homozygous rare[a] nonsynonymous variants | 10 |
| Homozygous rare presumed loss of function variants | 1 (in *TUB*) |

The data were also interrogated for homozygous rare variants predicted to affect pre-mRNA splicing; no variants were identified. [a] Presence in the 1,000 genomes data set with <0.5% minor allele frequency; the 20101123 sequence and alignment release including 1,094 individuals was used.

## Supp. Table S3. Genotypes and Phenotypes of Proband and Family Members

| Subject | Relationship to proband | Age (Yrs) | Genotype | BMI (kg/m$^2$) | Visual acuity RE LE | Refraction RE LE | Retinal appearance | OCT | FAF |
|---|---|---|---|---|---|---|---|---|---|
| II.4 (Proband) | - | 18 | Homozygous | 30 | 6/9<br>NPL | -1.25/-4.25,16<br>-1.00/-4.00,170 | RE: widespread RPE atrophy, generalised retinal pallor, arteriolar attenuation, fine peripheral pigmentary mottling, retinal white dots, macula spared, normal ON with PPA<br>LE: total RD | Preservation of the PR IS/OS junction at the fovea with drop off at the parafoveal region; inner retinal layers intact | Hyperautofluorescent annulus at fovea |
| II.3 | Brother | 21 | Homozygous | 23 | 6/18<br>6/18 | -3.00/+2.00, 90<br>-2.5/+1.75, 90 | BE: widespread RPE atrophy, generalised retinal pallor, arteriolar attenuation, fine peripheral pigmentary mottling, inferior retinal white dots, macula spared, normal ON with PPA | PR IS/OS layer preserved at the fovea; outer retinal debris at level of RPE in parafoveal region; inner retinal layers intact | Hypoautofluorescent mottling along vascular arcades but normal foveal autofluorescence signal |
| II.5 | Sister | 9 | Homozygous | 22* | 6/9<br>6/12 | +0.75/-3.50, 180<br>+0.25/-3.00, 180 | BE: mild peripheral RPE atrophy, generalised retinal pallor, fine inferior retinal pigmentary mottling, macula spared, normal ON with PPA | Normal | Normal |
| II.2 | Sister | 22 | Heterozygous | 20 | 6/5<br>6/5 | Plano<br>Plano | Normal | Not done | Not done |
| I.1 | Father | 47 | Heterozygous | 30 | 6/6 | Plano | BE: retinae and maculae normal, ON CDR 0.8 with inferior notching | Not done | Not done |

| Subject | Relationship to proband | Age (Yrs) | Genotype | BMI ($kg/m^2$) | Visual acuity | Refraction | Retinal appearance | OCT | FAF |
|---|---|---|---|---|---|---|---|---|---|
| | | | | | 6/6 | Plano | | | |
| I.2 | Mother | 45 | Heterozygous | 24 | 6/6 | Plano | Normal | Not done | Not done |
| | | | | | 6/6 | Plano | | | |

* BMI falls into the 98th centile for age and gender and is classed as obese; BE, Both Eyes; BMI, Body Mass Index; CDR, Cup – Disc Ratio; FAF, Fundus Autofluorescence; IS/OS, Inner segment / Outer Segment; LE, Left Eye; NPL, Nil Perception of Light; OCT, Optical Coherence Tomography; ON, Optic Nerve; PPA, Peripapillary Atrophy; PR, Photoreceptor; RD, Retinal Detachment; RE, Right Eye; RPE, Retinal Pigment Epithelium

**Supp. References**

Carr IM, Flintoff KJ, Taylor GR, Markham AF, Bonthron DT. 2006. Interactive visual analysis of SNP data for rapid autozygosity mapping in consanguineous families. Hum Mutat 27(10):1041-6.

Holder GE, Brigell MG, Hawlina M, Meigen T, Vaegan, Bach M. 2007. ISCEV standard for clinical pattern electroretinography--2007 update. Doc Ophthalmol 114(3):111-6.

Marmor MF, Fulton AB, Holder GE, Miyake Y, Brigell M, Bach M. 2009. ISCEV Standard for full-field clinical electroretinography (2008 update). Doc Ophthalmol 118(1):69-77.

Overlack N, Kilic D, Bauss K, Marker T, Kremer H, van Wijk E, Wolfrum U. 2011. Direct interaction of the Usher syndrome 1G protein SANS and myomegalin in the retina. Biochim Biophys Acta 1813(10):1883-92.

Plagnol V, Curtis J, Epstein M, Mok KY, Stebbings E, Grigoriadou S, Wood NW, Hambleton S, Burns SO, Thrasher AJ and others. 2012. A robust model for read count data in exome sequencing experiments and implications for copy number variant calling. Bioinformatics 28(21):2747-54.

Sergouniotis PI, Davidson AE, Mackay DS, Li Z, Yang X, Plagnol V, Moore AT, Webster AR. 2011. Recessive mutations in KCNJ13, encoding an inwardly rectifying potassium channel subunit, cause leber congenital amaurosis. Am J Hum Genet 89(1):183-90.

Trojan P, Krauss N, Choe HW, Giessl A, Pulvermuller A, Wolfrum U. 2008. Centrins in retinal photoreceptor cells: regulators in the connecting cilium. Prog Retin Eye Res 27(3):237-59.

Yang J, Liu X, Yue G, Adamian M, Bulgakov O, Li T. 2002. Rootletin, a novel coiled-coil protein, is a structural component of the ciliary rootlet. J Cell Biol 159(3):431-40.
